# Acute coronary syndrome and postprandial delayed hyperchylomicronemia

**DOI:** 10.18632/aging.101969

**Published:** 2019-05-14

**Authors:** Fumitaka Okajima, Osamu Kurihara, Masamichi Takano

**Affiliations:** 1 Division of Endocrinology, Nippon Medical School Chiba Hokusoh Hospital, Chiba, Japan; 2 Department of Endocrinology, Diabetes and Metabolism, Graduate School of Medicine, Nippon Medical School, Tokyo, Japan; 3 Cardiovascular Center, Nippon Medical School Chiba Hokusoh Hospital, Inzai, Chiba, Japan

**Keywords:** thin-cap fibroatheroma, optical coherence tomography, apolipoprotein B-48, apolipoprotein C-III, postprandial delayed hyperchylomicronemia

High low-density lipoprotein cholesterol (LDL-C) levels have been established as a risk factor for fatal cardiovascular (CV) events, including acute coronary syndrome (ACS), whose principal pathogenesis is the disruption of culprit plaques composed of a large lipid core under a thin fibrous cap. This structure, termed thin-cap fibroatheroma (TCFA), is considered vulnerable. Statins or 3-hydroxy-methylglutaryl coenzyme A reductase inhibitors can stabilize vulnerable coronary plaques by lowering LDL-C levels, and this stabilization prevents the occurrence of ACS. Clinical trials involving more than 170,000 participants conducted over two decades have demonstrated the beneficial effects of statins in reducing the risk of CV events by 25%; however, 75% of coronary events manifest despite treatment, possibly because of underlying residual risk [1].

The elevation of nonfasting serum triglyceride (TG) levels has been considered a residual risk factor for coronary artery disease (CAD) [2]. After food consumption, chylomicron (CM) and very-low-density lipoprotein (VLDL) are produced by the intestine and liver, respectively. After secretion, TGs contained in CM and VLDL particles are immediately hydrolyzed by lipoprotein lipase (LPL), and the resulting smaller CM and VLDL particles are termed remnants. CM remnants (CM-Rs) and a portion of VLDL remnants (VLDL-Rs) are removed from the circulation by the liver, and the remaining VLDL-Rs are transformed into LDL via hepatic TG lipase-induced hydrolyzation. CM, VLDL, CM-Rs, and VLDL-Rs are collectively termed triglyceride-rich lipoproteins (TGRLs). Postprandial hypertriglyceridemia is characterized by the accumulation of TGRL particles.

Apolipoprotein B-100 (ApoB-100) exists on the surface of VLDL, VLDL-R, and LDL, and apolipoprotein B-48 (ApoB-48) exists on the surface of CM and CM-R. ApoB-100 and ApoB-48 are the primary structural component of lipoproteins. Serum ApoB-100 levels mainly reflect the number of LDL particles opposed to VLDL or VLDL-R particles because the number of LDL particle is greater than that of VLDL or VLDL-R particles in serum. The serum levels of ApoB-48 reflect the number of CM and CM-R particles.

**Figure 1:**
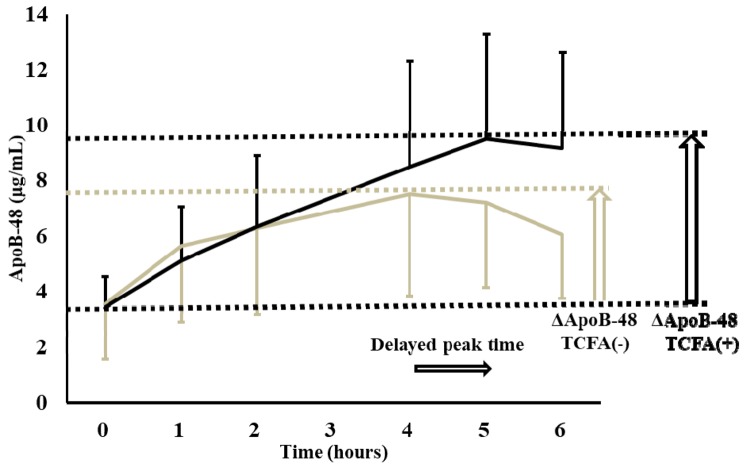
Increment of the plasma apolipoprotein B-48 (ApoB-48) from the fasting to peak values (ΔApoB-48) and peak time of ApoB-48 during meal tolerance test were significantly higher and delayed, respectively, in the patients with thin-cap fibroatheroma (TCFA) compared with the patients without TCFA [3].

Our recent study identified an independent association between the presence of TCFA detected via a multivessel examination using optical coherence tomography and the increment of serum ApoB-48 levels compared with the fasting to peak values (ΔApoB-48) estimated using the meal tolerance test (MTT) in patients with stable CAD who underwent percutaneous coronary intervention (Figure 1) [3]. Because the gradient of the mean ApoB-48 level within the first 2 h of MTT was the same, the increase in ΔApoB-48 and delay in the time to the peak value appeared to be associated with disturbance of hepatic CM clearance. In the same study, we also found an independent association between the presence of TCFA and fasting apolipoprotein C-III (ApoC-III) levels and a significant correlation between ΔApoB-48 and fasting serum ApoC-III levels.

ApoC-III is present on the surface of TGRLs together with ApoC-II. ApoC-II is an obligate activator of LPL [4]. Conversely, ApoC-III promotes the elevation of plasma TG levels by inhibiting the ApoC-II-induced activation of LPL. A recent study showed that ApoC-III also inhibits the hepatic clearance of TGRLs via the LDL receptor and LDL receptor-related protein 1 pathway [5]. Moreover, previous data illustrated that hydrolyzed ApoC-III-rich TGRLs are transformed into smaller-sized LDL particles called small, dense LDL, an especially atherogenic lipoprotein [6]. In our study, fasting ApoC-III levels and ΔApoB-48 were significantly correlated with TCFA as well as with each other. The elevation of ApoC-III levels is believed to delay and elevate postprandial CM metabolism and may increase the small, dense LDL levels.

Fibrates, which activate peroxisome proliferator-activated receptor alpha (PPARα), suppress the mRNA expression of ApoC-III and reduce serum ApoC-III levels as well as serum TG and ApoB-48 levels in the fasting and postprandial states. Fibrates are the most effective agents for lowering elevated TGRL levels. Prespecified and post-hoc subgroup analyses in a clinical trial using fenofibrate revealed a significant reduction in CV events in patients with high TG levels and low high-density lipoprotein cholesterol levels. Contrarily, the use of fibrates is frequently associated with adverse effects (AEs), such as an increase in liver enzyme and serum creatinine levels. Recently, pemafibrate, a novel selective PPARα modulator, has been developed. It induces AEs less frequently than fenofibrates [7]. The ongoing clinical trial “Pemafibrate to Reduce Cardiovascular OutcoMes by Reducing Triglycerides IN patiENts With diabeTes (PROMINENT),” which is assessing the effects of pemafibrate on CV outcomes, will clarify the efficacy and safety of the therapy.

Elevated serum ApoC-III levels might impair the postprandial metabolism of TGRLs and reduce the stability of coronary plaques. To prevent the incidence of ACS, reducing serum ApoC-III and TGRL levels in the fasting and postprandial states is an important and effective therapeutic strategy.
